# Modulation of Lung Immune Response 2014

**DOI:** 10.1155/2015/319593

**Published:** 2015-05-13

**Authors:** Alexandre de Paula Rogerio, Carlo José Freire Oliveira, Edinéia Lemos de Andrade, Troy Carlo

**Affiliations:** ^1^Departamento de Clínica Medica, Laboratório de Imunofarmacologia Experimental (LIFE), Instituto de Ciências da Saúde, Universidade Federal do Triângulo Mineiro (UFTM), Rua Vigário Carlos 162, 38025-350 Uberaba, MG, Brazil; ^2^Instituto de Ciências Biológicas e Naturais, Universidade Federal do Triângulo Mineiro (UFTM), 38025-180 Uberaba, MG, Brazil; ^3^Department of Pharmacology, Centre of Biological Sciences, Universidade Federal de Santa Catarina (UFSC), 88049-900 Florianópolis, SC, Brazil; ^4^Pulmonary and Critical Care Medicine Division, Brigham and Women's Hospital, Boston, MA 02115, USA

In recent decades, considerable progress has been made towards understanding the genetic, environmental, and immunological factors that contribute to the development of airway disorders. Current therapy for airway disorders has not advanced at the same pace as our increased understanding of the underlying causes of disease and is far from being ideal. This special issue places emphasis on the most relevant research into the pressing aspects of airway disorders including airway inflammation, tuberculosis, viral infection, and potential therapies.

Dietary changes due to our modern lifestyle have increased our susceptibility to inflammatory diseases such as airway inflammatory diseases. The Western diet (characterized by a high intake of omega-6 fatty acids and low intake of omega-3 fatty acids) is associated with unwanted proinflammatory effects and metabolic syndrome (including increased amounts of glucose and triglycerides circulating in the blood). On the other hand, diets rich in omega-3 fatty acids are associated with beneficial effects such as anti-inflammatory effects. Physical activity, another health related factor correlated with human lifestyle, can modulate the immune system with increased activity causing beneficial effects on systemic inflammation as well as overall health. To determine the effects of exercise and a high fat diet, S. P. Kurti et al. provided healthy volunteers with a high fat meal. Individuals were, next, randomly assigned to either a bout of moderate exercise or a rest period. Subjects were then tested for pulmonary function and samples were taken to determine the subjects metabolic and airway inflammatory states. The results demonstrated similar increases in dyslipidemia and markers of airway inflammation at 2 and 4 h after the meal regardless of exercise. These data led the authors to suggest that “it is necessary to develop other strategies to reduce the postprandial inflammatory burden on the airways that is present in asthmatics” and possibly in patients suffering from other airways inflammatory disorders.

Acute inflammation is generally self-limited. However, if acute inflammation fails to resolve, chronic inflammation can persist. The innate and adaptive immune systems, as well as structural cells, modulate the length and intensity of inflammatory responses. As cited above, lipids mediators, derived from the omega-6 polyunsaturated fatty acids (PUFA) including leukotrienes (LTs) and prostaglandins (PGs), are potent enhancers of innate and adaptive immune activity and are implicated in numerous inflammatory disorders. On the other hand, lipids mediators, derived from the omega-3 polyunsaturated fatty acids (PUFA), including resolvins, maresins, and protectins, dampen inflammation and promote resolution. J. R. de Oliveira et al. demonstrated the effect of aspirin-triggered-resolvin D1 (AT-RvD1, the R carbon 17 epimer of resolvin D1) on bronchial epithelial cells (BEAS-2B) stimulated with IL-4, a Th2 cytokine involved in the modulation of allergic airway inflammation such as asthma. AT-RvD1 blocked the IL-4-triggered activation of bronchial epithelial cells. AT-RvD1 exposure, signaling through the ALX/FPR2 receptor, led to decreased CCL2 (involved in eosinophilic inflammation) and CXCL-8 (involved in neutrophilic inflammation) protein levels and downregulated both NF-*κ*B and STAT6 pathways. In addition, AT-RvD1 decreased SOCS1 and increased SOCS3 RNA levels. These results suggest that AT-RvD1 can inhibit the neutrophilic and eosinophilic airway inflammation associated with asthma.

The immunoglobulin E (IgE) can be produced by body upon exposure to allergens such as pollen and is associated with allergic disorders such as atopic dermatitis (eczema), urticaria, asthma, and others. IgE can bind to mast cells through high-affinity immunoglobulin (Ig) E receptors (Fc*ε*RI). Upon re-exposure, the binding of the allergen to IgE present on mast cells/basophils leads to degranulation and the release of proinflammatory mediators such as cytokines, leukotrienes, chemokines, and proteases involved in the modulation of the pathophysiology of allergic diseases. Omalizumab is a recombinant humanized monoclonal antibody that prevents IgE from interacting with the FC*ɛ*RI on mast cells/basophils thereby blocking the release of soluble proinflammatory mediators. In an in-depth review, A. D. Yalcin describes our current understanding of the effectiveness of omalizumab in several diseases such as hyperimmunoglobulin-E syndrome, eosinophilic gastroenteritis, mastocytosis, pruritic bullous pemphigoid, nasal polyps, atopic dermatitis, chronic urticaria, and others.

Tuberculosis is a global public health problem with an enormous social impact. Upon interaction with* Mycobacterium tuberculosis,* the immune system releases a number of cytokines (such as IFN-*γ*, IL-4, and IL-17) which are secreted by specific immune cells (Th1, Th2, and Th17 helper T cells, resp.). Th1 and Th17 cytokines provide essential signals for the control of* M. tuberculosis* infection while Th2 cytokines antagonize the protective effects of Th1 and Th17 cytokines and result in unregulated* M. tuberculosis* infection. Higher concentrations of IL-17 and IFN-*γ* are found in bacillus Calmette-Guérin (BCG) vaccinated and clinically cured tuberculosis patients compared to those patients with active infection. In contrast, infected patients display higher concentration of IL-4 than the other groups. Regulatory T cells (Tregs) also play an important role in* M. tuberculosis* infections. They regulate immune responses by limiting the extent of the immune response; however, this activity may also facilitate chronic infection and block microbial eradication. M. V. Silva et al. isolated peripheral blood mononuclear cells (PBMCs) from three groups of volunteers, healthy individuals with no history of tuberculosis but with a positive tuberculin skin test (healthy control), patients with clinically cured pulmonary tuberculosis (TB-treated group), and patients with active pulmonary tuberculosis (TB-active group). The isolated PBMCs were stimulated with mycobacterium antigens extracted from* M. bovis* (bacillus Calmette-Guérin (BCG)) and characterized the CD4^+^ T cells as Th1 (T-Bet^+^ and IFN-*γ* ^+^), Th2 (GATA-3^+^ and IL-4^+^), Th17 (ROR*γ*T^+^ and IL-17^+^), or Treg (using CD25 and FoxP3 to distinguish subtypes). The results as described by the authors demonstrated that the active TB patients showed a global reduction of T helper cells but a ratio favoring Th2 and Treg populations, whereas cured TB patients display increased subpopulations of CD4^+^ T cells (Th1, Th2, Th17, and Treg), although Th1 and Th17 cells predominate suggesting that changes in the repertoire of mycobacterium-specific Th cells are associated with clinical cure after treatment of pulmonary tuberculosis.

Viruses such as rhinoviruses, paramyxoviruses, coronaviruses, and influenza viruses are major causes of human respiratory tract diseases. Respiratory viruses damage lung structural cells and trigger a host inflammatory response inflammation that can lead to further tissue injury. Severe injury to lung epithelial cells lowers the efficiency of alveolar gas-exchange processes and can result in hypoxia, respiratory failure, and death. Respiratory syncytial virus (RSV) is a major cause of bronchiolitis and severe acute respiratory infections and may become lethal. Risk factors such as low gestational age, low birth weight, and malformations correlate with the chances of severe RSV infection in the airways. There is no specific treatment for RSV infection making the understanding of the genetic and immunological factors critical for the development of future therapies. In this issue, S. Vandini et al. review critical aspects of RSV infections including environmental and host factors influencing virus activity, the elicited immune response, and the actions of nonimmunological factors. In addition, they provide information regarding animal models of RSV infection and their limitations.

